# Simultaneous gastric, pancreatic, and renal metastasis from poorly differentiated hepatocellular carcinoma

**DOI:** 10.1007/s12328-024-01935-w

**Published:** 2024-03-07

**Authors:** Manabu Uchikoshi, Takayoshi Ito, Yoko Nakajima, Ikuya Sugiura, Shoujirou Uozumi, Yuu Shimozuma, Masashi Sakaki, Yasuyuki Ohira, Toshiko Yamochi, Hitoshi Yoshida

**Affiliations:** 1 Division of Gastroenterology, Department of Internal Medicine, Showa University School of Medicine, 1-5-8 Hatanodai, Shinagawaku, Tokyo 142-8666 Japan; 2 Digestive Diseases Center, Showa University Koto Toyosu Hospital, 5-1-38 Toyosu, Kotoku, Tokyo 135-8577 Japan; 3 Department of Pathology and Laboratory Medicine, Showa University School of Medicine, 1-5-8 Hatanodai, Shinagawaku, Tokyo 142-8666 Japan

**Keywords:** Hepatocellular carcinoma, Extrahepatic metastasis, Adenocarcinoma

## Abstract

Common extrahepatic metastasis sites of hepatocellular carcinoma (HCC) are the lungs, adrenal glands, and bones. Herein, we report a rare case of metastatic gastric, pancreatic, and renal tumors from HCC simultaneously, and review the relevant literature. A 75-year-old woman presented with right hypochondralgia, appetite loss, and weight loss. Computed tomography revealed suspected metastatic liver, lung, and renal tumors. A blood test revealed a leukocyte count of 26,210/μL and a high inflammatory reaction. As sepsis was suspected, the patient was referred to our hospital. Gadolinium–ethoxybenzyl–diethylenetriamine pentaacetic acid-enhanced magnetic resonance imaging revealed a hypovascular liver tumor that was suspected to be metastatic. Upper gastrointestinal endoscopy revealed two suspected metastatic gastric tumors. Liver and gastric tumor biopsies revealed poor carcinoma in both. The patient’s condition gradually worsened and she died on day 8 of the illness. Based on autopsy findings, the patient was finally diagnosed with metastatic gastric and renal tumors originating from HCC. Additionally, a metastatic pancreatic tumor originating from the HCC was identified during autopsy. The pathological diagnosis of the pulmonary lesion was primary lung adenocarcinoma. In conclusion, HCC should be suspected in cases with multiple metastases of unknown primary lesions.

## Introduction

Hepatocellular carcinoma (HCC) is the most common malignant liver cancer. In recent times, the number of patients with HCC has decreased due to the development of new treatments; however, HCC remains the fourth most common cause of cancer-related deaths worldwide [1]. Approximately 50% of the HCCs are caused by hepatitis C virus infection. The next most common causes of HCC are hepatitis B virus infection, autoimmune hepatitis, and non-alcoholic steatohepatitis. Extrahepatic metastases of HCC to the lungs, adrenal glands, and bones are frequently observed. Hepatic resection is the first-line treatment for a few HCCs without extrahepatic metastasis; however, transcatheter arterial chemoembolization (TACE) is indicated for multiple HCCs. Chemotherapy is generally selected for cases in which extrahepatic metastasis is observed. Gastric, pancreatic, and renal metastases from HCC are extremely rare. In this report, we describe the case of a patient with poorly differentiated HCC who developed concurrent metastatic gastric, pancreatic, and renal tumors.

## Case report

**Fig. 1:**
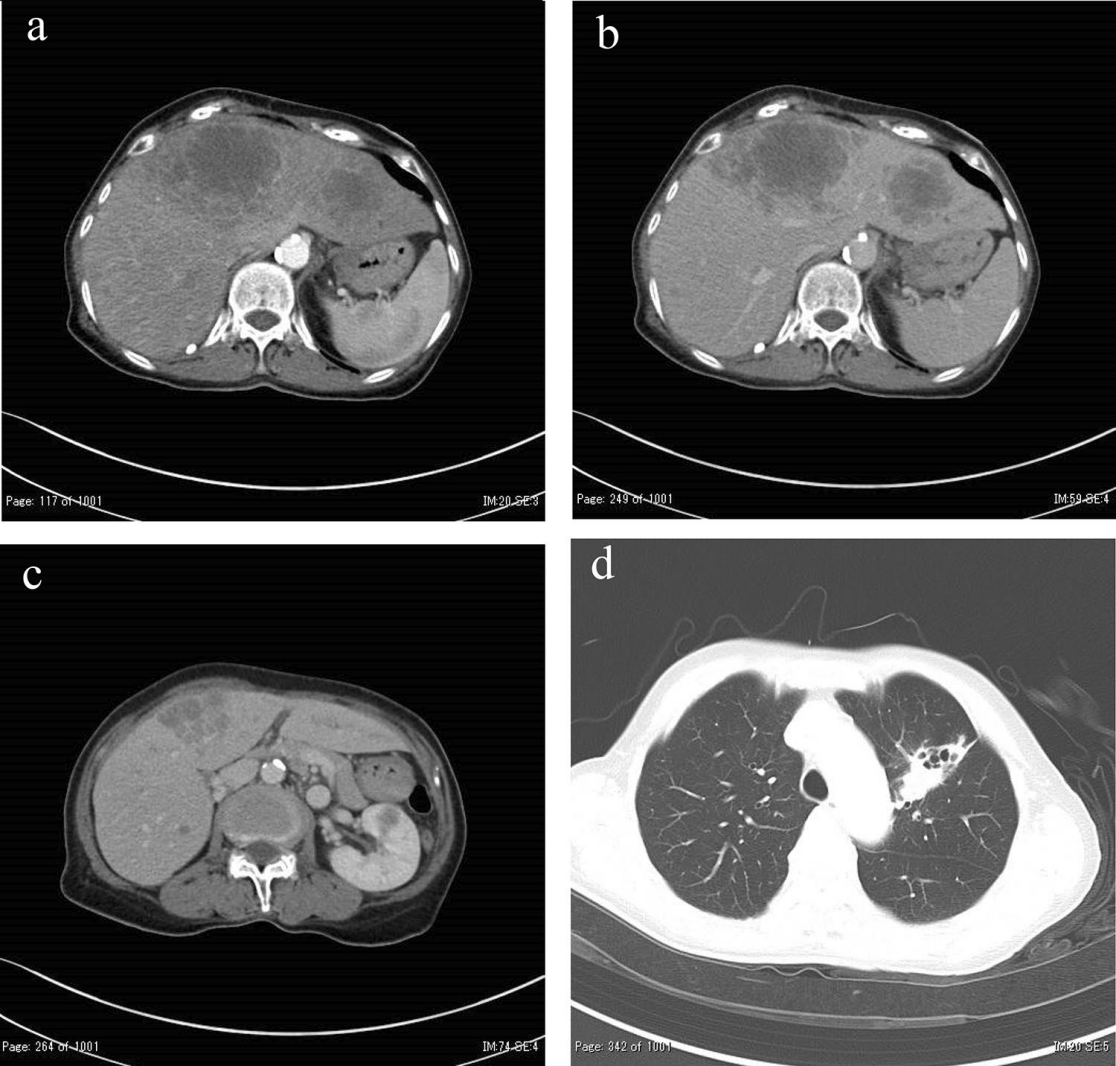
Contrast-enhanced computed tomography of chest and abdominal region. **a** Liver tumor at arterial phase of enhancement. **b** Liver tumor at portal phase of enhancement. There was faint contrast around the tumor, and almost contrast effect was not observed in the center of the tumor. **c** Renal tumor. The tumor was faintly contrasted, but early deep staining was not observed. **d** Lung tumor at pulmonary window setting. The nodular shadow with a cavity was observed in the left upper lung

**Fig. 2:**
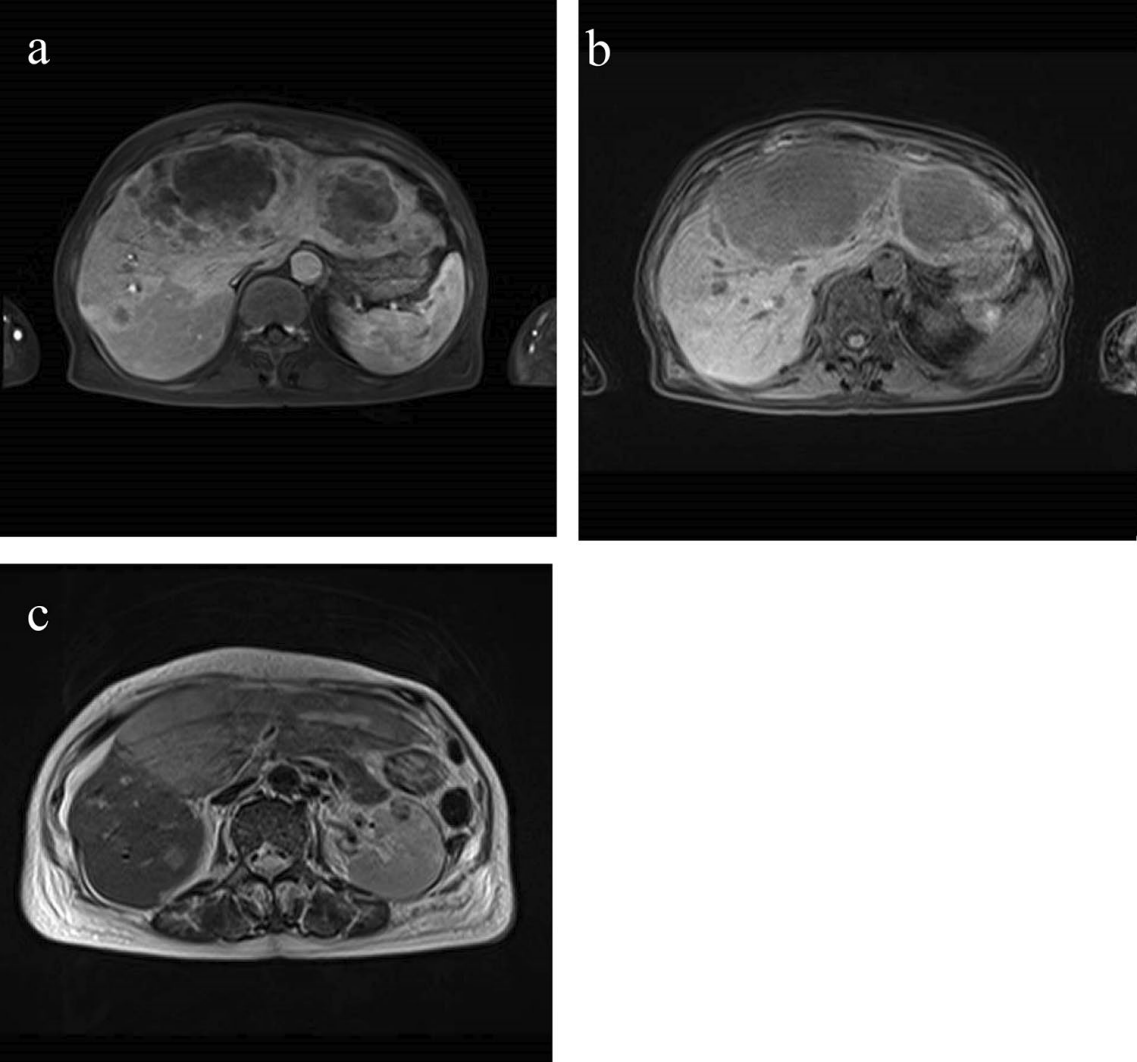
Gadolinium–ethoxybenzyl–diethylenetriamine pentaacetic acid (Gd–EOB–DTPA)-enhanced magnetic resonance imaging of abdominal region. **a** Liver tumor at arterial phase of enhancement. **b** Liver tumor at hepatobiliary phase. There was faint contrast around the tumor, and almost contrast effect was not observed in the center of the tumor same as CT scan. **c** Renal tumor. The tumors with poor contrast enhancement was observed

**Fig. 3:**
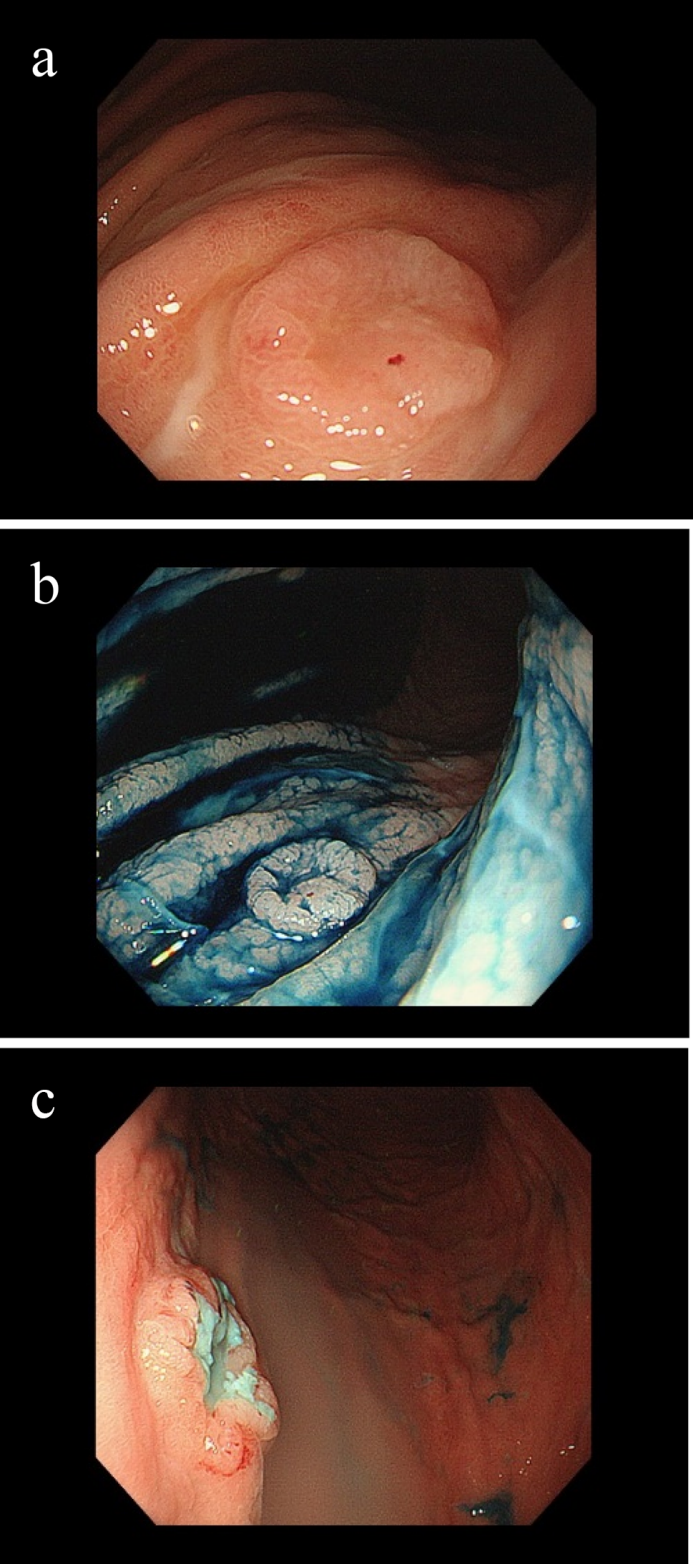
The gastrointestinal endoscopy at the admission. **a** Gastric tumor at the greater curvature of upper gastric body. **b** Gastric tumor at the greater curvature of upper gastric body (Indigo carmine spraying). **c** Gastric tumor at the anterior wall of upper gastric body

**Fig. 4:**
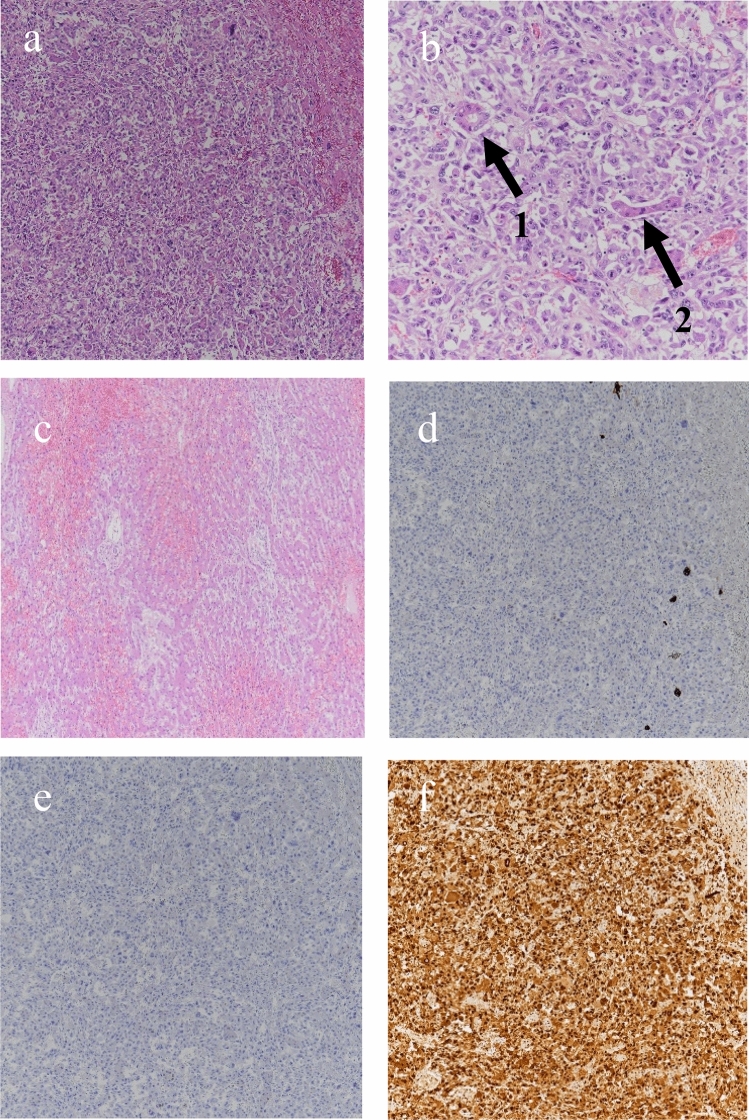

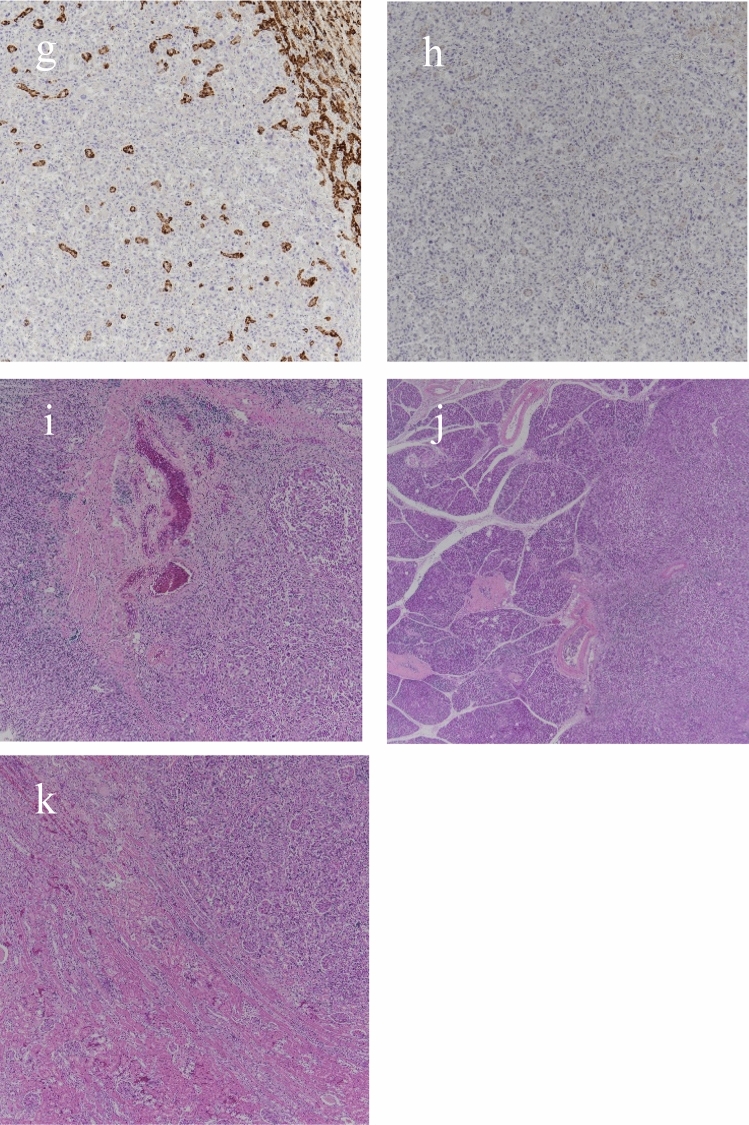
Pathological findings of liver, gastric, pancreatic, renal tumor, and background liver. **a** Liver (HE ×40). Cobblestone-like growth of atypical cells with enlarged nuclei with distinct nucleoli. **b** Liver (HE ×100). Arrow 1: Pseudo-glandular pattern in liver tissue. Arrow 2: Cord-like structure in liver tissue. **c** Liver (Background: HE ×40). Liver congestion was observed, but almost no fibrosis or inflammation was observed. **d** Liver (CK7 ×40). The liver tumor was negative for CK7 immunostaining. **e** Liver (CK20 ×40). The liver tumor was negative for CK20 immunostaining. **f** Liver (HSP70 ×40). The liver tumor was strong positive for HSP70 immunostaining. **g** Liver (TTF-1 ×40). The liver tumor was negative for TTF-1 immunostaining. **h** Liver (Napsin A ×40). The liver tumor was negative for Napsin A immunostaining. **i** Stomach (HE ×20). Poorly differentiated tumor tissue was proliferating, but no tumorous glandular tissue was observed. **j** Pancreas (HE ×10). Poorly differentiated tumor tissue was proliferating, but no tumorous glandular tissue was observed. **k** Kidney (HE ×20). Growth of cancerous atypical cells around the glomerulus was observed

The patient was a 75-year-old woman who presented with primary complaints of right hypochondralgia, appetite loss, and weight loss. The patient had a history of hypertension and spinal caries. She was informed of liver damage 3 years ago but did not undergo a medical examination in a hospital. She had no history of smoking or drinking. She experienced mild right hypochondralgia, appetite loss, and weight loss from the beginning of May in Year X. Because the pain had exacerbated, she consulted a nearby doctor at the end of May in year X. Following a blood test and computed tomography (CT) at a hospital, sepsis, metastatic liver, lung, and renal tumors were suspected. Thus, the patient was transferred to our hospital for further examination and treatment. Physical examination at hospitalization revealed slight tachycardia, jaundice of the palpebral conjunctiva, and tenderness of the right hypochondriac region. Other physical examinations revealed no remarkable findings. She underwent contrast-enhanced CT, gadolinium–ethoxybenzyl–diethylenetriamine pentaacetic acid (Gd–EOB–DTPA)-enhanced magnetic resonance imaging (MRI), and blood examination at our hospital to establish a diagnosis. Laboratory findings are shown in Table 1. Laboratory data revealed high white blood cell count (23.4 × 10^3^/μL) and C-reactive protein levels (19.9 mg/dL), indicating inflammation. Serum aspartate aminotransferase, alanine aminotransferase, alkaline phosphatase, γ-glutamyl transferase, and lactate dehydrogenase levels were within the normal ranges (14, 15, 289, 177, and 152 IU/L, respectively). Tests for hepatitis B surface antigen and hepatitis C antibodies were negative. Levels of tumor markers, including α-fetoprotein, des-gamma-carboxy prothrombin, carcinoembryonic antigen, and carbohydrate antigen 19–9, were within the normal ranges (<2 ng/ml, 18.5 mAU/ml, 1.5 ng/ml, and 3.7 U/ml, respectively). Contrast-enhanced CT of the chest, abdomen, and pelvis revealed multiple low-attenuation areas, with comparatively poor contrast in the liver and kidney. The maximum diameter of the liver tumor was approximately 18 cm. In addition, the cavernous lesion of the left lung superior lobe exhibited tuberculosis; however, the result of the acid-fast bacterial culture was negative, suggesting the possibility of a tumor (Fig. 1). Gd–EOB–DTPA-enhanced MRI revealed multiple liver tumors. Because there were multiple tumors in the liver and the surrounding area of the tumor was faintly contrasted and necrosis was observed in the center, it was initially suspected that it was a metastatic tumor rather than a primary tumor. In addition, the renal tumors were faintly enhanced and multiple, indicating that they were due to metastasis (Fig. 2). Endoscopy was performed to check for primary lesions in the multiple tumor sites. Upper gastrointestinal endoscopy revealed atrophic gastritis and two suspected regions of metastasis in the stomach. The size of one lesion at the anterior wall of the upper gastric body was 18 mm, and the size of another lesion at the greater curvature of the upper gastric body was 12 mm. In addition, the 18 mm lesion was crater-shaped (Fig. 3). We performed a gastric biopsy under endoscopy and a liver biopsy under ultrasonography to confirm the diagnosis. Pathological examinations of both the liver and stomach tumors showed poorly differentiated carcinomas. The pathologists at our hospital considered immunostaining to be more definitive for a final report. At this point, the liver, kidney, and stomach tumors were suspected to be metastases; however, the primary lesion of these tumors was not clear on contrast-enhanced CT and Gd–EOB–DTPA-enhanced-MRI. The patient’s high fever, hypercalcemia, and hypoalbuminemia gradually worsened. Antibiotics were administered, but the patient showed symptoms of hypotension and a decreased level of consciousness before a definitive diagnosis could be made. The patient died on day 8 of the illness before the final pathological report was completed. A pathological autopsy was performed at the wishes of the patient’s family. The results of immunostaining for the liver, stomach, pancreas, and kidney were negative for cytokeratin 7, cytokeratin 20, α-fetoprotein, and glypican 3; however, these tissues revealed a pseudo-glandular pattern and a cord-like structure, which are characteristic of HCC (Fig. 4). The pulmonary lesion tested positive for thyroid transcription factor-1 (TTF-1) and Napsin A, which were characteristic of lung adenocarcinoma. Furthermore, when immunostaining of the liver tumor was additionally performed, immunostaining for TTF-1 and Napsin A was negative, but immunostaining for HSP70 was strongly positive. Immunostaining for CK7, CK20, TTF-1, and Napsin A was negative in the tumor tissues of the stomach, pancreas, and kidney, and as no other primary tumor was detected, it was considered to be metastasis of hepatocellular carcinoma. There was no evidence of chronic liver disease in non-cancerous liver tissue. There was little background inflammation or fibrosis in the liver, only pericentral venous congestion reflecting general condition and poor hemodynamics. Regarding the final report of the pathological autopsy, the primary lesion was a poorly differentiated HCC. The tumors located in the stomach and kidney were the metastasis of the HCC. In addition, metastatic foci of HCC were found in the pancreas for the first time during the pathological autopsy. The pulmonary tumor was not a metastasis of the HCC but an isolated adenocarcinoma.

**Table 1:** Laboratory examination on admission

CBC		Biochemistry		Tumor marker	
WBC	23,400/μl	TP	5.7 g/dl	AFP	<2 ng/ml
RBC	316 × 10^4^/μl	Alb	2.0 g/dl	DCP	18.5 mAU/ml
Hb	9.6 g/dl	T-Bil	2.2 mg/dl	CEA	1.5 ng/ml
Plt	27.6 × 10^4^/μl	D-Bil	0.6 mg/dl	CA19-9	3.7 U/ml
		AST	14 IU/l		
Coagulation test		ALT	15 IU/l	Serological test	
PT (%)	56	ALP	289 IU/l	CRP	19.9 mg/dl
PT (INR)	1.34	LDH	152 IU/l	HBsAg	<2.0 S/N
FDP	44.0 μg/dl	γ-GTP	177 IU/l	HCVAb	<1.0 S/CO
d-Dimer	19.1 μg/ml	AMY	23 mg/dl		
		FBS	141 mg/dl		
		BUN	19.0 mg/dl		
		Cre	0.39 mg/dl

## Discussion

Characteristics of HCC progression includes growth of HCC, intrahepatic metastasis, distant metastasis, and dissemination. Extrahepatic metastasis of HCC has been reported to occur frequently (30–75%) [2]. Distant HCC metastases usually occur in the lungs, adrenal glands, and bones. Metastasis of HCC to the stomach is extremely rare and reportedly has a prevalence of 4–12% [3, 4]. However, direct invasion was assumed in these cases. We estimated that there were fewer cases of stomach metastases than previously reported. It was reported that only 6 of 48,473 patients with solid tumors had gastric metastasis of HCC [5]. A PubMed search for gastric metastasis of HCC resulted in only nine cases [6–14]. Regarding metastasis to the stomach, two major mechanisms are considered. The first mechanism involves the portal vein circulatory system. Tumor cells flow into the circulatory system from the collateral circulation developed in liver cirrhosis and engraft into the stomach wall. The second mechanism involves the portal retrograde system. The tumor cells flow backward in the left gastric vein owing to the tumor thrombus and are implanted into the vein of the gastric wall. The major complications of gastric metastasis are gastrointestinal bleeding (84%), abdominal pain (23%), and gastrointestinal obstruction (16%); however, these were not observed in this case [15]. Although the prognosis of gastric metastasis of HCC is poor, surgical resection may improve patient prognosis.

Metastatic pancreatic tumors originating from HCC are extremely rare. According to Reddy et al., the incidence of metastatic pancreatic tumors among all pancreatic malignant tumors is less than 2% [16]. Furthermore, the prevalence of metastatic pancreatic tumors originating from HCC is considered to be <1% [17]. We conducted a literature survey of pancreatic metastasis of HCC in PubMed and found only five cases [18–22]. Metastasis to the pancreas is often isolated, and distinguishing between primary and metastatic pancreatic tumors is difficult using imaging alone; therefore, pathological diagnosis is often necessary. Generally, surgery and chemotherapy are selected as treatment modalities following pathological diagnosis. Surgical treatment is often performed for pancreatic metastatic tumors; however, its prognosis is generally poor [23]. Therefore, chemotherapy is often recommended for primary and metastatic pancreatic tumors. In this case, the pancreatic metastatic lesion was small (<10 mm); thus, it could not be confirmed using CT or MRI and was only observed during pathological autopsy.

Furthermore, renal metastasis is also rare, with a prevalence of 7.2% based on autopsy findings in patients with malignant tumors [24]. Primary tumors of the lungs, breasts, and gastrointestinal tract are the most common origins of renal metastasis; renal metastases from HCC are exceedingly rare. In a literature search using PubMed, we identified only six cases of renal metastasis from HCC [25–30]. Tumor markers such as α-fetoprotein and imaging findings may be helpful for diagnosis in these cases; tumor rupture was also observed in some cases. Although there is no consensus regarding the optimal treatment strategy, surgery is often selected when there are no other distant metastases, and chemotherapy is often selected when there are distant metastases.

In this case, the background liver was almost normal, and the cause of hepatocellular carcinoma development is unclear. It has been reported that 7.9% of all hepatocellular carcinomas originate from normal livers, and the present case was considered to fall under this category [31].

In summary, we report the case of a 75-year-old woman with concurrent gastric, pancreatic, and renal metastases of HCC. As gastric, pancreatic, and renal metastases of HCC are very rare, this case with three simultaneous metastases is of clinical interest. Additionally, primary lung adenocarcinoma was observed. Unfortunately, in this case, the patient’s condition worsened rapidly and she died before treatment could be initiated. Pathological autopsy revealed that the lesions in the stomach, pancreas, and kidney were metastases of HCC. We believe that HCC should be considered as a differential diagnosis if simultaneous gastric, pancreatic, and renal metastases from an unknown primary lesion are observed.

## References

[1] Bray F, Ferlay J, Soerjomataram I (2018). Global cancer statistics 2018: GLOBOCAN estimates of incidence and mortality worldwide for 36 cancers in 185 countries. CA Cancer J Clin.

[2] Lin CP, Cheng JS, Lai KH (2000). Gastrointestinal metastasis in hepatocellular carcinoma: radiological and endoscopic studies of 11 cases. J Gastroenterol Hepatol.

[3] Anthony PP (1973). Primary carcinoma of the liver: a study of 282 cases in Ugandan Africans. J Pathol.

[4] Nakashima T, Okuda K, Kojiro M (1983). Pathology of hepatocellular carcinoma in Japan. 232 Consecutive cases autopsied in ten years. Cancer..

[5] Wu MH, Lin MT, Lee PH (2007). Clinicopathological study of gastric metastases. World J Surg.

[6] Peng L, Yu K, Li Y (2018). Gastric metastasis of recurrent hepatocellular carcinoma: a case report and literature review. J Cancer Res Ther.

[7] Haruki K, Misawa T, Gocho T (2016). Hepatocellular carcinoma with gastric metastasis treated by simultaneous hepatic and gastric resection: report of a case. Clin J Gastroenterol.

[8] Abdul Hakim MS, Azmi AN, Jayalakshmi P (2018). Gastric metastasis from hepatocellular carcinoma: a rare manifestation. J Gastrointest Cancer.

[9] Sakumura M, Tajiri K, Sugiyama T (2018). Gastric metastasis of hepatocellular carcinoma mimicking early gastric cancer. Clin Gastroenterol Hepatol.

[10] Imai M, Ishikawa T, Okoshi M (2019). Hemorrhagic gastric metastasis from hepatocellular carcinoma successfully treated using coil embolization of the left gastric artery. Intern Med.

[11] Hu ML, Tai WC, Chuah SK (2010). Gastric metastasis of hepatocellular carcinoma via a possible existing retrograde hematogenous pathway. J Gastroenterol Hepatol.

[12] Li L, Zhang WH, Meng FP (2015). Gastric metastasis of hepatocellular carcinoma with gastrointestinal bleeding after liver transplant: a case report. Transplant Proc.

[13] Inagaki Y, Shiraki K, Takei Y (2014). Gastric metastasis of hepatocellular carcinoma presenting as hematemesis. Clin Gastroenterol Hepatol.

[14] Qian L, Huang J, Qin H (2014). Glypican-3-expressing gastric metastasis of hepatocellular carcinoma via curative subtotal gastrectomy: a case report. J Gastrointest Cancer.

[15] Lin TL, Yap AQ, Wang JH (2011). Long term survival in patients with hepatocellular carcinoma directly invading the gastrointestinal tract: case reports and literature review. Surg Oncol.

[16] Reddy S, Wolfgang CL (2009). The role of surgery in the management of isolated metastases to the pancreas. Lancet Oncol.

[17] Klein KA, Stephens DH, Welch TJ (1998). CT characteristics of metastasic disease of the pancreas. Radiographics.

[18] Woo SM, Park JW, Han SS (2010). Isolated pancreatic metastasis of hepatocellular carcinoma after curative resection. World J Gastrointest Oncol.

[19] Zhang Y, Han T, Wang D (2019). Hepatocellular carcinoma with pancreatic mass as the first symptom: a case report and literature review. Ann Palliat Med.

[20] Xiong J, Kwong Chian S, Li J (2017). Iodine-125 seed implantation for synchronous pancreatic metastases from hepatocellular carcinoma: a case report and literature review. Medicine.

[21] Nishiofuku H, Marugami N, Tanaka T (2013). Isolated fat-containing pancreatic metastasis from hepatocellular carcinoma. Jpn J Radiol.

[22] Sugai Y, Watanabe Y, Hasoya T (1999). Pancreatic metastasis from hepatocellular carcinoma. AJR Am J Roentgenol.

[23] Roland CF, van Heerden JA (1989). Nonpancreatic primary tumors with metastasis to the pancreas. Surg Gynecol Obstet.

[24] Bracken RB, Chica G, Johnson DE (1979). Secondary renal neoplasms: an autopsy study. South Med J.

[25] Yamanaka R, Sekino Y, Babasaki T (2020). Renal metastasis from primary hepatocellular carcinoma: a case report. Int Cancer Conf J.

[26] Aron M, Nair M, Hemal AK (2004). Renal metastasis from primary hepatocellular carcinoma. A case report and review of the literature. Urol Int.

[27] Kinoshita O, Ichijo Y, Yoneda M (2017). Spontaneous rupture of renal metastasis from hepatocellular carcinoma. Case Rep Surg.

[28] Ong KW, Joseph B, Gyomber DV (2013). Nephrectomy for a renal metastasis of undiagnosed hepatocellular carcinoma arising from an orthotopic liver transplant undertaken for cryptogenic cirrhosis. Korean J Urol.

[29] Hsu YB, Lee PH, Sheu JC (1994). Hepatocellular carcinoma with metastasis to the kidney: report of a case. J Formos Med Assoc.

[30] Shiozaki T, Hayakawa K, Tanikake M (2003). Accumulation of 99mTc-PMT in renal metastasis of hepatocellular carcinoma. Ann Nucl Med.

[31] kudo M, Izumi N, kokudo N (2016). The Japan Liver Cancer Association 19th National Primary Liver Cancer Follow-up Survey report.

